# Adherence to Drug-Refill Is a Useful Early Warning Indicator of Virologic and Immunologic Failure among HIV Patients on First-Line ART in South Africa

**DOI:** 10.1371/journal.pone.0017518

**Published:** 2011-03-09

**Authors:** Ziad El-Khatib, David Katzenstein, Gaetano Marrone, Fatima Laher, Lerato Mohapi, Max Petzold, Lynn Morris, Anna Mia Ekström

**Affiliations:** 1 Division of Global Health (IHCAR), Karolinska Institutet, Stockholm, Sweden; 2 AIDS Virus Research Unit, National Institute for Communicable Diseases (NICD), Johannesburg, South Africa; 3 Division of Infectious Diseases, Stanford University, Palo Alto, California, United States of America; 4 Perinatal HIV Research Unit (PHRU), University of the Witwatersrand, Soweto, South Africa; 5 Nordic School of Public Health (NHV), Gothenburg, Sweden; University of Cape Town, South Africa

## Abstract

**Background:**

Affordable strategies to prevent treatment failure on first-line regimens among HIV patients are essential for the long-term success of antiretroviral therapy (ART) in sub-Saharan Africa. WHO recommends using routinely collected data such as adherence to drug-refill visits as early warning indicators. We examined the association between adherence to drug-refill visits and long-term virologic and immunologic failure among non-nucleoside reverse transcriptase inhibitor (NNRTI) recipients in South Africa.

**Methods:**

In 2008, 456 patients on NNRTI-based ART for a median of 44 months (range 12–99 months; 1,510 person-years) were enrolled in a retrospective cohort study in Soweto. Charts were reviewed for clinical characteristics before and during ART. Multivariable logistic regression and Kaplan-Meier survival analysis assessed associations with virologic (two repeated VL>50 copies/ml) and immunologic failure (as defined by WHO).

**Results:**

After a median of 15 months on ART, 19% (n = 88) and 19% (n = 87) had failed virologically and immunologically respectively. A cumulative adherence of <95% to drug-refill visits was significantly associated with both virologic and immunologic failure (p<0.01). In the final multivariable model, risk factors for virologic failure were incomplete adherence (OR 2.8, 95%CI 1.2–6.7), and previous exposure to single-dose nevirapine or any other antiretrovirals (adj. OR 2.1, 95%CI 1.2–3.9), adjusted for age and sex. In Kaplan-Meier analysis, the virologic failure rate by month 48 was 19% vs. 37% among adherent and non-adherent patients respectively (logrank p value = 0.02).

**Conclusion:**

One in five failed virologically after a median of 15 months on ART. Adherence to drug-refill visits works as an early warning indicator for both virologic and immunologic failure.

## Introduction

Antiretroviral treatment (ART) has saved millions of lives by transforming HIV infection from a fatal into a chronic disease [1] and the vast majority (97%) of patients in sub-Saharan Africa receive a non-nucleoside reverse transcriptase (NNRTI) based regimen as first-line treatment [2]. Given the costs associated with second-line protease inhibitors, the long-term sustainability of ART in many of low- and some middle-income countries depends on finding feasible ways for early detection of treatment failure to maintain patients on first-line regimens [3], [4].

Since viral load (VL) monitoring is not currently accessible in most resource-limited high-endemic contexts [2], [5], patients are often continued on first-line ART until the emergence of clinical symptoms or until any of the World Health Organization (WHO) criteria for immunologic failure are met. Although virologic failure and drug resistance usually precede immunologic failure, these measures are not always well correlated at clinical follow-up [6], [7], [8]. To routinely assess the effectiveness of ART at HIV treatment clinics and to minimize preventable HIV drug resistance (HIVDR), WHO recommends using available site-based data from medical and pharmacy records, e.g. on-time adherence to monthly ART drug pick-up and clinic appointment-keeping [9], [10], as an early warning indicator (EWI) of inconsistent drug exposure. Failure to pick up drugs on time serves as a proxy for treatment interruption and suboptimal drug concentrations, which are associated with virologic failure and the evolution of drug resistance [11], [12], [13].

Earlier reports from resource-limited settings have defined virologic failure as a VL of >400, >1,000 or >5,000 copies/ml at one or two repeated visits [14], [15]. However, drug resistance mutations can emerge at lower VL levels [16] and in high-income countries, monitoring guidelines recommend using VL >50 copies/ml as an indicator of virologic failure for patients on ART [17]. As more robust, sensitive and lower cost assays are developed, ART programmes in low- and middle-income may be able to adopt lower threshold values for virologic failure. Thus, we assessed the proportion of repeated VL >50 copies/ml, immunologic failure and median CD4 cell count gains in a cohort of long-term, first-line recipients in Soweto, who had been on NNTRI-based regimens for up to eight years. We also assessed the relationship between cumulative adherence to drug-refill visits and treatment failure.

## Methods

### Setting and study population

At the time of the study, more than 1,500 patients were receiving ART at this clinic. Aiming for a precision of +/− 5% for a proportion of 50% using 95% confidence interval, a sample size of 384 patients was required [18]. Adding a margin to the minimum required sample size, a cohort of 458 participants on long-term ART (range 12 to 99 months) were enrolled during March-September 2008 [19] for a cross-sectional assessment and a retrospective review of their medical charts. The inclusion criteria were: being on NNRTI-containing regimen for ≥12 months; consent to an interviewer-mediated questionnaire; medical record review; and withdrawal of 10ml of blood. The study, named South African Virologic Evaluation (SAVE) [15], was performed at the Perinatal HIV Research Unit (PHRU) adult HIV clinic at the Chris Hani Baragwanath Hospital, in the Soweto township outside Johannesburg, South Africa. The study site [20] and a cross-sectional description of viremia and drug resistance have been described in depth previously [15], [21].

### Data collection

Patients were interviewed at study enrolment to obtain demographics, socioeconomic characteristics, reasons for missing doses and the strategies used to remember taking their pills on time. The retrospective review included: year of HIV diagnosis; pre-ART initiation characteristics (VL, CD4 cell count, pre-exposure to single-dose nevirapine (sdNVP) or other antiretroviral drugs (ARVs); current and previous TB therapy; dates for drug refill visits; and treatment interruptions. VL and CD4 counts had been measured every six months on average. For two of the patients, medical records were not available and they were excluded from the longitudinal analysis, leaving 456 patients for data analyses. A survey form, called Medical Records-SAVE (MR.SAVE©), was developed, piloted and modified [22] for data collection. EpiData [23], [24] was used for data entry.

### Definitions

The clinic uses a private diagnostic laboratory service that provides VL measures with the Versant HIV-1 RNA 3.0 (Siemens Deerfield, IL, USA) based on bDNA technology, with a lower limit of detection of 50 copies/ml. Virologic failure was defined as two repeated VL >50 copies/ml at any time after three months on ART. Immunologic failure was defined according to the WHO guidelines as having either (i) a CD4 cell count of <100 cells/µl post six months on ART, (ii) a CD4 cell count of less or equal to CD4 pre-ART after six months on ART or (iii) >50% reduction from the on-ART peak CD4 cell count [25]. Patients were dispensed ART monthly and at scheduled doctor's visits. They were also given projected monthly pharmacy refill dates and the date of the next doctor's appointment. At each refill visit, pharmacy staff dispensed pills and recorded the date. To estimate adherence, we calculated the total number of days that the patient was late for the drug-refill visits divided by the total duration on ART. The formula was: [The number of days late for drug-refill visit  =  (Date when the patient came for drug refill - Date of the pre-scheduled appointment indicated on the patient's medical record)]. The results were then summarized for repeated refill visits to obtain the cumulative number of days coming late per client. To estimate adherence, the following formula was used [The cumulative number of days coming late ×100)/Total number of days the patient was assumed to be exposed to ART given the dispensed number of pills]. Appointment dates were censored after the date of virologic failure.

Incomplete versus complete adherence was defined as a cumulative adherence to drug refill visits of < and ≥95% respectively. Treatment interruption was defined as a reported and/or planned history of stopping and resuming therapy, identified through chart review.

### Statistical analysis

Descriptive analyses including median (IQR) for numerical variables, frequencies and proportions for categorical variables were performed. Bivariate analyses to assess risk factors for virologic and immunologic failure were performed using Pearson Chi Square and Fisher's exact tests. Thereafter variables with a p-value ≤0.10 were added into a multivariate logistic regression model and those with a p-value <0.05 were considered significant in the final multivariate model, calculating odds ratios (OR) and 95% confidence intervals (CIs). However, the variables sex and age were always maintained in the final multivariate models to account for possible remaining confounders.

Kaplan Meier survival analysis was done using months as the time unit in order to assess time to virologic and immunologic failure on ART among all patients.

Known pre-ART risk factors, exposure to sdNVP [26], [27], [28] or any type of ARVs, CD4 cell count and age [26], [29], [30] were adjusted using Cox regression analysis for virologic failure. For immunologic failure, Cox regression analysis was done by adjusting for the same above-mentioned variables and any virologic failure. Due to collinearity between sex and pre-ART CD4 count, sex was not included in the final survival model.

Finally, we assessed the median gain in CD4 cell count during ART on a six-monthly basis (range +/−3 months) among 1) patients with incomplete vs. complete cumulative drug-refill adherence, and 2) patients with virologic failure vs. suppression up to 36 months on ART only, due to data availability.

Stata/SE College Station, Texas (version 10.1) [31] and Graphpad Prism (version 4.0c) [32] were used for data analysis. In the survival analysis, the command sts graph in STATA was used. Adjusting for covariates, the command fits separate Cox regression models for each group, and the separately calculated baseline survivor was then retrieved.

Ethical approval for this study was obtained from the research ethics committees at the University of the Witwatersrand in Johannesburg, South Africa, and the Regional Medical Ethics Board in Stockholm, Sweden. Written informed consent was obtained from all patients.

## Results and Discussion

### Patients' characteristics

A total of 456 patient records were reviewed. Most patients (79%) were diagnosed with HIV between 2001-2004 (median 2003), 14% before 2000 and the remaining 7% between 2005 and 2008. Pre-ART initiation, 51% (222/434) had a CD4 cell count of ≤100 cell/µl and 41% (172/421) had a VL of ≥100 000 copies/ml (Table 1). Overall, the median time on ART was 44 months (IQR 38–48; 1,510 person-years) and 77% (349/456) were women (Table 1). Eighteen percent (80/445) had been exposed to ARVs previously; 15% of the women (52/349) had received sdNVP for the prevention of mother-to-child transmission (PMTCT) with a median time before ART initiation of 15 months, and 6% of all patients (28/446) had received ART before starting the current ART regimen. Approximately half (48%, 199/414) had been treated for tuberculosis (TB) before ART initiation. At the time of study enrolment, the patients spent a median of 40 minutes (IQR 30–60) travelling to the clinic by mini-van (90%), walking (5%) and using their own car (4%).

**Table 1:** Demographics, socioeconomic and clinical characteristics and bivariate analysis for the association with a) virologic and b) immunologic failure among 456 patients on ART in Soweto, South Africa.

Demographics and socio-economic characteristics		a) Virologic failure∧		b) Immunologic failure∧∧	
	N = 456# (% of total)	N = 88/456 (% row)	Bivariate p-value	N = 87/456# (% row)	Bivariate p-value
**Sex**					
Women	349 (77%)	68 (19%)		59 (17%)	
Men	107 (23%)	20 (19%)	0.87	28 (26%)	0.03
**Age**					
18–24	21 (5%)	5 (24%)	0.42	3 (14%)	0.62
25–34	235 (51%)	45 (19%)		46 (20%)	
35–44	147 (32%)	24 (16%)		25 (17%)	
≥45	53 (12%)	14 (26%)		13 (24%)	
**Marital status**					
Have partner	265 (58%)	49 (18%)		50 (19%)	
Single/no partner at all	191 (42%)	39 (20%)	0.61	37 (19%)	0.89
**Born in South Africa**					
No	17 (4%)	4 (23%)		2 (12%)	
Yes	439 (96%)	84 (19%)	0.65	85 (19%)	0.43
**Education level**					
No education or primary schooling	50 (11%)	11 (20%)	0.61	4 (8%)	
Secondary or tertiary education level	406 (89%)	77 (19%)		83 (20%)	0.03*
**Year diagnosed with HIV**					
≤2000	63 (14%)	10 (16%)	0.79	10 (16%)	0.66
2001–2004	348 (79%)	68 (20%)		70 (20%)	
2005–2008	31 (7%)	6 (19%)		5 (16%)	
**Any type of work**					
No	283 (65%)	59 (21%)		60 (21%)	
Yes	154 (35%)	28 (18%)	0.51	23 (15%)	0.11
**Clinical characteristics** **					
**CD4 cell count – pre-ART**					
≤50	122 (28%)	21 (17%)	0.59	26 (21%)	0.52
51–100	100 (23%)	24 (24%)		21 (16%)	
101–249	199 (46%)	37 (19%)		31 (16%)	
≥250	13 (3%)	2 (15%)		3 (23%)	
**Viral load level – pre-ART (RNA copies/ml)**					
<5,000	22 (5%)	2 (9%)	0.54	1 (5%)	0.26
5,000–29,999	110 (26%)	23 (21%)		24 (22%)	
30,000–99,999	117 (28%)	20 (17%)		24 (21%)	
≥100,000	172 (41%)	35 (20%)		30 (17%)	
**Pre-exposure to sdNVP or other type of ARVs**					
Treatment naïve	365 (82%)	64 (18%)		72 (20%)	
Single dose-nevirapine (sdNVP)	52 (12%)	13 (25%)	0.19	7 (13%)	0.28
Had any ARVs pre-ART initiation##	28 (6%)	9 (32%)	0.06	6 (21%)	0.83
**Treatment experienced (sdNVP or any ARVs)**	80 (18%)	22 (28%)	0.04	13 (16%)	0.47
**TB therapy**					
Not treated before ART	215 (52%)	36 (17%)		38 (18%)	
Treated before ART	137 (33%)	27 (20%)	0.48	33 (24%)	0.14
Was on TB therapy when started on ART	62 (15%)	11 (18%)	0.85	10 (16%)	0.78
**Disclosed HIV status pre-ART**					
No	35 (8%)	4 (11%)		8 (23%)	
Yes	400 (92%)	80 (20%)	0.22	77 (19%)	0.61
**Cumulative adherence to on-time drug refill**					
95-100%	430 (94%)	79 (18%)		78 (18%)	
<95%	26 (6%)	9 (35%)	0.04	9 (35%)	0.04
**Any treatment interruption**					
No	324 (71%)	66 (20%)		58 (18%)	
Yes$	132 (29%)	22 (17%)	0.36	29 (22%)	0.32

*2-sided Fisher exact test;

**All patients were initiated on an NNRTI-based regimen (the majority had efavirenz);

# Two patients (2/458) were excluded due to missing longitudinal data;

∧Two repeated VL >50 copies/ml post-three months on ART;

∧∧WHO criteria for immunologic failure;

## Two patients were exposed to sdNVP and ART, pre-ART initiation, but virologically suppressed.

$ Treatment interruption: once for 129 patients and twice for 3 patients.

### Virologic failure

Overall, 19% (88/456) met the criteria for virologic failure. In bivariate analysis, being exposed to any type of ARVs including sdNVP prior to ART initiation or incomplete adherence was significantly associated with virologic failure (p = 0.04) (Table 1a). These two factors remained significant after adjustment for confounding by age and sex in the multivariable analysis model. The odds ratio of virologic failure among patients with incomplete adherence almost tripled (adjusted OR 2.8, 95%CI 1.2–6.7) and doubled among those exposed to any type of ARVs prior to ART initiation (adj. OR 2.1, 95%CI 1.2–3.9). However, exposure to sdNVP alone did not reach statistical significance. Neither the level of CD4 cell count nor VL at pre-ART initiation was significantly associated with subsequent virologic failure on ART in bivariate analyses, and therefore was not included in the multivariable model.

In a separate analysis, there was no significant association between incomplete adherence and any of the patients' demographic, socioeconomic or clinical data (not shown).

Planned treatment interruptions were not part of the clinical guidelines at this clinic. Of the 456 patients, 132 (29%) had a reported history of treatment interruption registered in their medical charts, 98% of these (129/132) only once. Three patients were reported to have experienced treatment interruption twice but were not found to fail virologically during the study period. The median duration of treatment interruption was longer among patients with, compared to those without, virologic failure; 45 days (IQR 27–97) vs. 36 days (IQR 19–63) respectively, although this difference was not statistically significant (p = 0.12).

**Figure 1:**
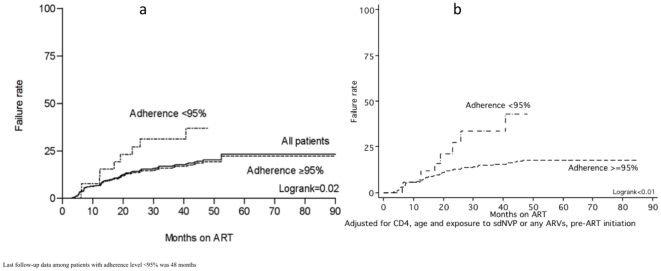
(a) Kaplan-Meier survival analysis for time to virologic failure by level of cumulative adherence to drug refill visits. (b) Cox regression analysis after adjustment for confounding by CD4 cell count, age and being exposed to sdNVP or any ART pre-ART initiation.

Analysing the risk of virologic failure over time using Kaplan Meier survival analysis, the virologic failure rate was 23% up until month 99 (Figure 1a). There was a significant difference in time to virologic failure between patients with complete vs. incomplete adherence. By month 12 on ART, the failure rate was similar (7% vs 8% among patients with complete and incomplete adherence respectively) but by month 48, the difference in failure rates had reached statistical significance between the groups, 19% vs. 37% respectively (logrank p value = 0.02) (Figure 1a). Following adjustment for CD4 cell count, age and exposure to any ARVs pre-ART initiation in a Cox regression analysis, there was a significant difference in virologic failure rates already at month 12 on ART; 2% vs 11%, and at month 48. This difference was even more pronounced, 18% vs. 43%, among patients with complete vs incomplete adherence respectively (logrank p value <0.01) (Figure 1b).

### Immunologic failure

Overall, 87/456 (19%) of the patients met one or more of the definitions of immunologic failure based on CD4 cell count [33]. In bivariate analysis, immunologic failure was associated with incomplete adherence (p = 0.04), gender (p = 0.03), and low education level (p = 0.03) (Table 1). However, none of these variables remained significant in the final multivariate logistic regression model. More than one third (37%; 32/87) of patients failing immunologically were also found to be viremic. Among those with immunologic failure, there was no significant difference in CD4 cell count or VL, pre-ART initiation, between the 32 viremic and 55 non-viremic patients (data not shown).

**Figure 2:**
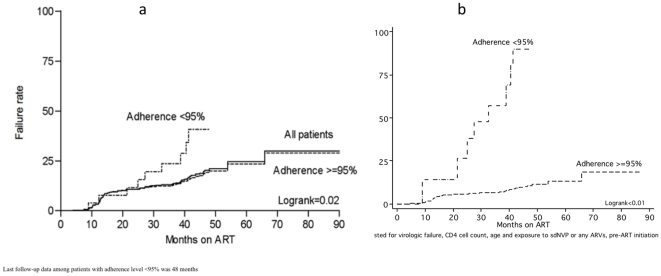
(a) Kaplan-Meier survival analysis for time to immunologic failure, by level of cumulative adherence to drug refill visits. (b) Cox regression analysis, adjusted for confounding by virologic failure, CD4 cell count, age and being exposed to sdNVP or any ART pre-ART initiation

Kaplan Meier survival analysis demonstrated an overall immunologic failure rate of 27% by month 48 (Figure 2a). The risk of immunologic failure was 41% vs 19% among those with incomplete and complete adherence respectively after 48 months on ART (logrank p value = 0.02). After adjustment for CD4 cell count, age and exposure to any ARVs pre-ART initiation and for virologic failure in the Cox regression analysis, patients with incomplete adherence had an immunologic failure rate of 90% at month 48, while the corresponding figure among patients with complete adherence was only 11% (Figure 2b) (logrank p value<0.01).

### Immunologic response to ART

**Figure 3:**
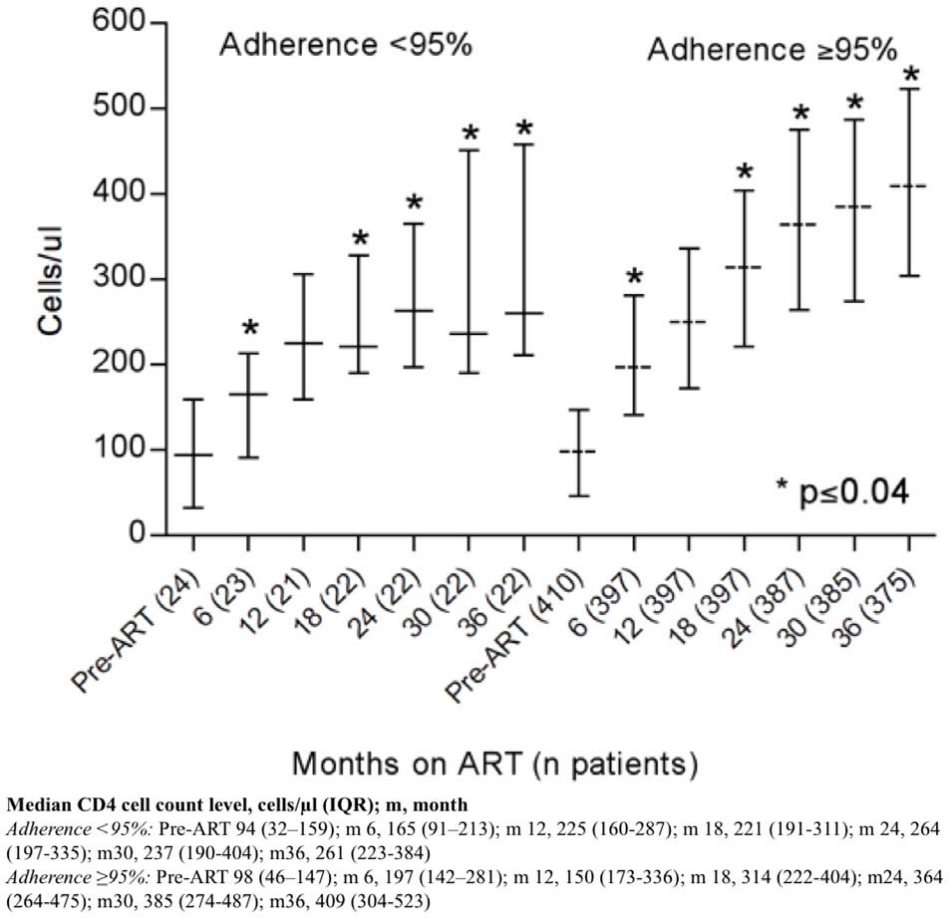
Median CD4 (IQR) for patients initiated on ART, by adherence < or ≥95% based on drug-refill visits.

**Figure 4:**
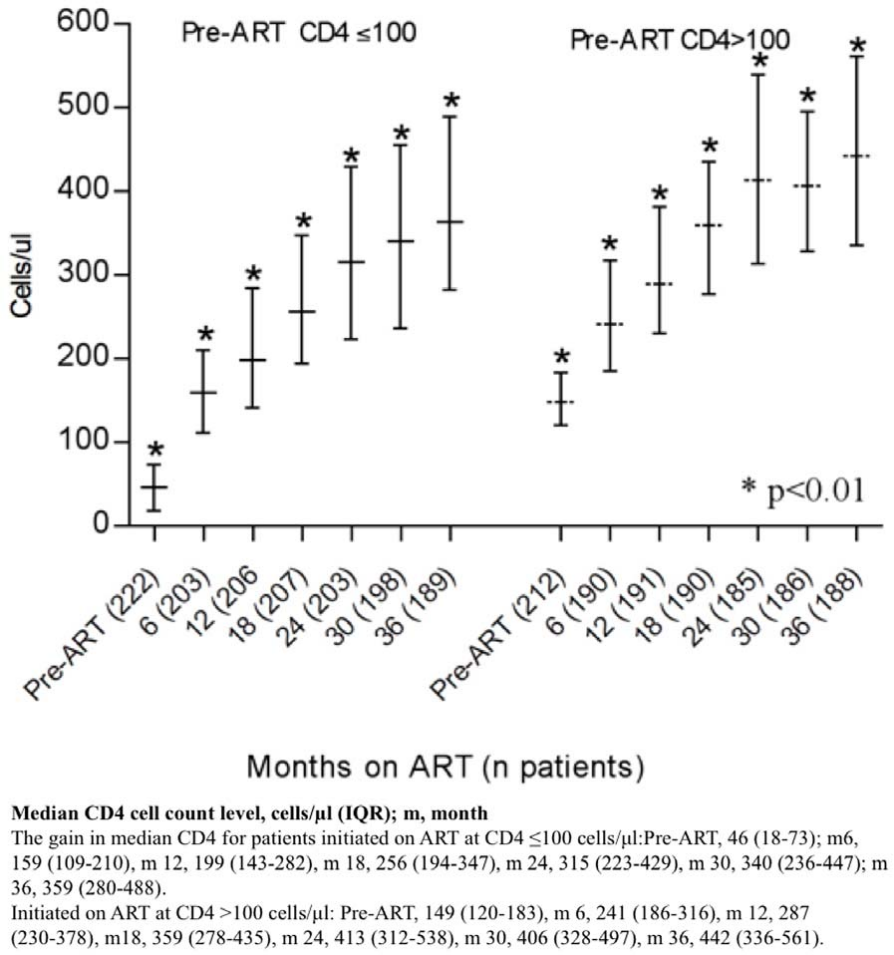
Median CD4 (IQR) for patients initiated on ART, with CD4 ≤ or >100 cells/µl.

**Figure 5:**
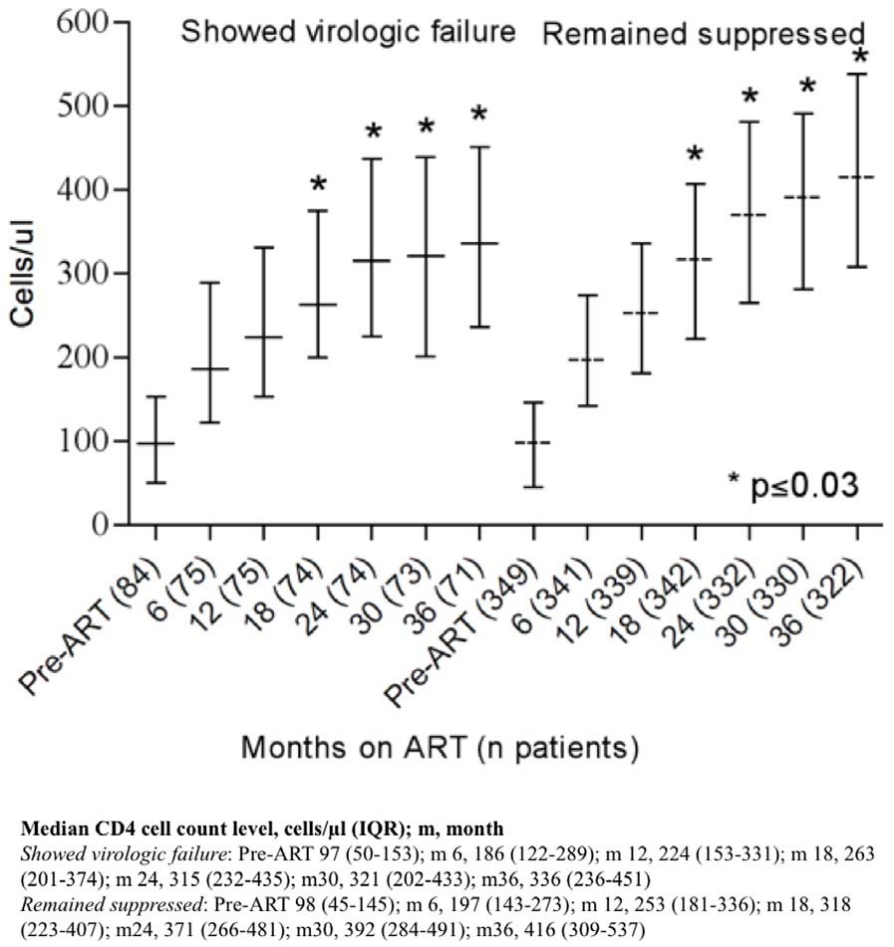
Median CD4 (IQR) for patients who showed virologic failure vs. those who remained suppressed.

There was a significant difference in CD4 cell count gain during ART between patients with complete vs incomplete adherence at almost all of the clinical visits (p≤0.04) (Figure 3). As expected, patients initiating ART with a CD4 cell count of >100 cells/µl had a significantly higher median CD4 cell count in comparison to patients started on ART at a CD4 cell count of ≤100 cells/µl throughout the follow-up period (Figure 4). The median CD4 cell count was also significantly higher among patients with continuous virologic suppression patients as compared to those with virologic rebound up to at least three years on ART (months 18, 24, 30 and 36, p≤0.03) (Figure 5).

### Barriers and facilitators for adherence

**Figure 6:**
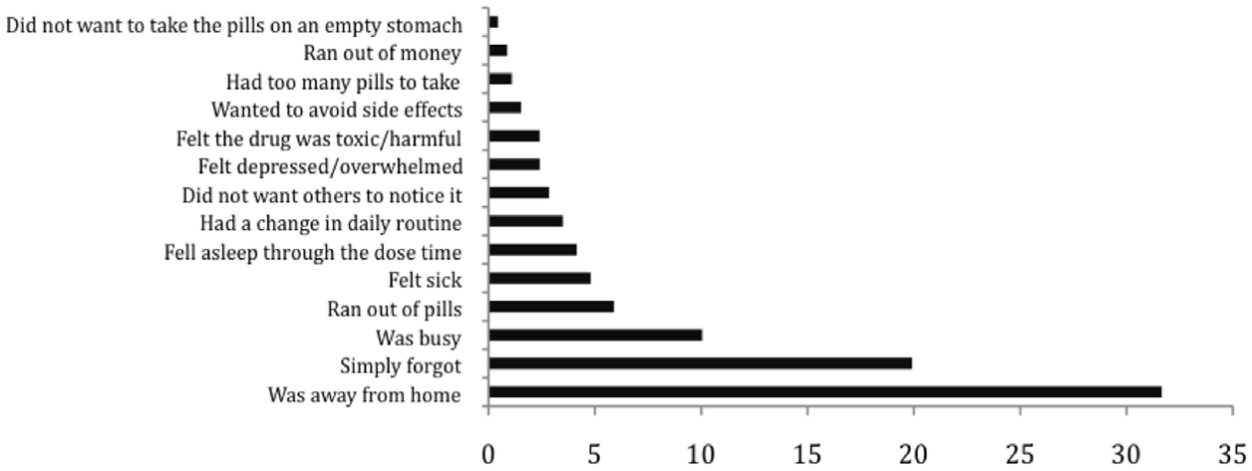
Proportion of patients (%) with self-reported reasons for not taking any of their pills in general at study enrolment (N = 458).

When all patients were asked about the reasons for missing pills, the three main reasons stated were: being away from home (32%); simply forgetting (20%); and being busy with other things (10%) (Figure 6). Eleven other reasons were reported but all with a prevalence of ≤6%.

Patients used a combination of methods to remember their medication; mobile phone alarms (49%), relying on their own memory (49%), a close friend/relative (20%) or a partner (8%) to remind them, or pill-boxes (1%).

All interviewees denied any intentional non-adherence in order to maintain eligibility for disability support. This was despite 39% of the patients reporting that their primary source of financial support was the social welfare disability grant provided in South Africa for HIV-infected patients with a CD4 cell count below 200 cells/µl.

Antiretroviral therapy is a life-long undertaking and finding feasible and affordable means for early detection of treatment failure is crucial to sustain first-line therapy effectiveness. Our study found that the estimated proportion of patients failing virologically was 2–3 times higher among patients who were late for their drug-refill visits compared to those with an adherence to drug refill above 95%. This provides evidence that failure to collect ART may serve as a proxy for reduced drug exposure over time.

According to WHO [9], [10], monitoring the extent to which ART sites function through EWIs such as adherence to on-time drug refills is of high priority in order to minimize preventable HIVDR. The usefulness and importance of the on-time drug refill indicator became most obvious in the Cox regression analysis over time. It showed a significant difference in first-year failure rates (2% vs 11%) among patients with complete adherence vs incomplete adherence to drug refill already after 12 months on treatment. This difference became even more visible after 4 years on ART (18% vs 43% virologic failure rate respectively) adjusting for CD4 cell count at ART initiation, age and exposure to any ARVs pre-ART initiation.

The cumulative proportion with virologic failure in our study is comparable to similar cohorts of long-term ART-recipients in urban South African townships including other studies from an urban site in Johannesburg [34], and from Khayelitsha [30]. Similarly high failure rates have also been reported from Switzerland [35], England [36], [37], France, Spain, Germany and Canada [37].

Apart from <95% adherence to drug refills, previous exposure to other ARVs was the only significant risk factor associated with an increased risk of virologic failure in our multivariable analysis, supporting ample existing evidence that sdNVP [38], [39], [40], [41] or other ARVs [42] may predispose to virologic failure and the emergence of HIVDR mutations among women treated with NNRTI-based ARVs.

During the first year on ART, CD4 T-cell restoration can be slow [43], but we found that the rate of CD4 cell count gain up to 36 months on ART was the same regardless of the initial level of CD4. Thus, patients starting ART with advanced immuno-suppression and very low CD4 cell counts (<100 cells/µl) maintained a significantly lower CD4 cell count level throughout the study period. This put them at risk of increased morbidity for a number of years post-ART initiation [30], [44], and provides further justification to initiate ART earlier, as embodied in the recent WHO recommendations [33]. The gain in CD4 cell count was significantly dependent on high adherence to drug refill and viral suppression supporting the findings by Bisson and co-workers [45]. However, in our study, nearly two thirds of patients failing immunologically were virologically suppressed at clinical assessment. Thus CD4 cell count is a poor predictor of virologic outcomes [6], [7], [46], [47] and the use of immunological criteria only for monitoring treatment responses may jeopardize clinical management.

All study participants in this study had been on ART for at least 12 months, and over 90% had achieved a CD4 cell count of >200 cells/µl. There is an ongoing debate in South Africa where some have suggested that HIV patients may keep their CD4 counts at <200 cells/µl by intentionally missing doses [48], [49] to retain eligibility for disability grants. However, this was firmly denied by our participants, who rather ascribed missing doses to being away from home or simply forgetting to take their pills. The interesting fact that half of our patients used their mobile phones as a medication reminder opens up future opportunities of systematic adherence support through text reminders since cell phone access and usage are rapidly increasing throughout sub-Saharan Africa [50]. Additionally, dispensing ART for more than 30 days at a time may also reduce the risk of missed doses.

VL measurements are rarely available in most resource-limited settings and where measured, virologic failure has often been defined as VL >400, >1,000 or even >5,000 copies/ml [15], [29], [30]. In this study, a more sensitive and conservative definition for virologic failure was used, >50 copies/ml. While the fact that some patients with intermittent viremia (blips) were included may cause some concern, 85% (75/88) were confirmed viremic in two sequential assessments >50 copies/ml. In this study, we used <95% as a conservative cut-off point for incomplete adherence. In early adherence studies, including unboosted protease inhibitors (PIs), a 95% adherence level was shown to be associated with high virologic suppression [51], a greater increase in CD4 cell count, and lower hospitalization rates [52], [53]. More recent studies have indicated that moderate adherence levels (70–90%) may be enough to achieve acceptable virologic suppression with antiretroviral regimens containing boosted PIs or NNRTIs. However, at the 95% level we can be quite certain that a very small proportion of individuals on NNRTI are likely to fail virologically [54], [55], [56]. The aim of the current study was to look at treatment failure among long-term recipients of ART, i.e. excluded those with <12 months of ART [15]. However, our retrospective assessment of medical charts went back and traced all virologic failures, including the first year on treatment, enabling us to assess the risk of failure from treatment start-up to a maximum follow-up of over 8 years. However, with this design, we missed those who first failed virologically and then dropped out of the programme before reaching 12 months on treatment. Given that a substantial proportion of patients enrolled in ART in similar urban settings are expected to drop out early [57], this would lead to an underestimation of the true virologic failure rate among NNRTI recipients in the current assessment.

Finally, the lack of a significant association between longer documented treatment interruptions and virologic failure is likely due to incomplete clinical data since we only had access to reported interruptions recorded in the medical charts.

In summary, there was a strong association between cumulative reduced adherence to drug-refill visits and both virologic and immunologic failure. One in five failed virologically, the majority within the first two years on ART. On-time ART pick-up appears to be a feasible tool to identify individuals at risk, and if followed by prompt and targeted interventions, it could be used to reduce the rate of virologic failure.
